# Production and Properties of Starch: Current Research

**DOI:** 10.3390/molecules29030646

**Published:** 2024-01-30

**Authors:** Lili Wang, Litao Tong

**Affiliations:** Institute of Food Science and Technology, Chinese Academy of Agricultural Sciences, Haidian District, Beijing 100193, China

## 1. Introduction

Starch is an important carbohydrate polymer found in plants and has been widely used in food and non-food industries due to its abundance, renewability, biodegradability, low cost, biocompatibility and non-toxicity [1]. The functional properties of starch are variable due to its different sources and structures [2]. Factors such as starch granule morphology, amylose to amylopectin ratio, molecular structure, degree of branching in terms of steric hindrance and, consequently, mass transfer resistance can affect the physicochemical properties and application of starch [3,4,5]. Native starch has been modified by various processes for desired industrial applications over the last few decades [6,7]. There has been intense interest in developing novel methods, with may present advantages of high efficiency, environmental friendliness and easier starch production and modification [8]. Although more attention is paid to the functional properties of modified starch for practical applications in the industry, structural changes are the basis of these functional changes; hence, understanding the structural alterations induced by processing techniques is a fundamental subject when considering the better utilization of starch and starch modification techniques. The current research is also geared towards the importance of starch in human nutrition and health. Different forms of starch in foods and novel food processing, with respect to its digestibility, have received tremendous research focus [9] as the microstructure of starch has a great influence on its digestibility [10]. Starch multi-scale structures, including the amylose/amylopectin ratio, fine structures of amylose and amylopectin, short-range ordered structures, helical structures, crystalline features, lamellar structures and morphology, are factors that determine enzyme binding and catalyzation with starch in the human diet [11,12,13]. This Special Issue introduces the latest research, using a variety of raw materials for analysis, on the physical and chemical properties of starch after modification, intrinsic structure changes after extraction and modification methods, and starch’s digestive and anti digestive properties. It contains ten articles and a review; we will provide a brief overview of the contents of this Special Issue in the following paragraphs. On this matter, we clarify that it is not the purpose of this Editorial to elaborate on each of the texts, but rather to encourage the reader to explore them. 

## 2. An Overview of Published Articles

Hesham Alqah et al. (Contribution 1) discuss how starches from different botanical sources are affected in the presence of enzymes. This study investigated the impact of α-amylase on the water holding capacity, freezable water content, sugar content and water sorption isotherm of pregelatinized starches derived from chickpea, wheat, corn, white beans and sweet potatoes. The results show that the water holding capacity and the sugar content of starch increased significantly when the annealing temperature and GSE were increased. The ordering of starches in terms of their freezable water content is as follows: chickpea starch > white beans starch > wheat starch > chickpea starch > sweet potato starch. The moisture sorption of different pregelatinized starches increased with increasing water activity at different annealing temperatures and was characterized as Type II isotherm. The equilibrium moisture content and monolayer moisture contents decreased after treatment with 0.1 mL GSE but increased after 1.0 mL GSE treatment and longer annealing time. This study demonstrated that the cost of the analyzed procedure is significantly lower in comparison to utilizing refined α-amylase extract.

The article by Nie et al. (Contribution 2) investigated the gelatinization and retrogradation properties of highland barley starch (HBS) using different extraction methods. In this paper, the effect of different extraction methods on starch was explained by obtaining HBS through three methods: alkali extraction (A-HBS), ultrasonic extraction (U-HBS) and enzyme extraction (E-HBS), which were used to explain the apparent morphology, structural changes and physicochemical properties of the extracted starch. The results showed that A-HBS- and U-HBS-treated starch had less surface damage and the E-HBS starch had a rough surface, but none of them changed the integrity. Compared to the other two extraction methods, A-HBS showed the weakest hydrogen bonding, which resulted in the highest viscosity, and could, thus, be used as a food thickener. The different extraction methods did not change the crystalline type of HBS, but E-HBS had the highest relative crystallinity value; thus, it exhibited better thermal stability, which increased the crispness of foods such as cookies. U-HBS had the highest damaged starch content, as well as the lowest gelatinization enthalpy, after 1 and 7 days of storage, respectively, providing anti-aging properties which make it suitable for foods with a short shelf life such as bread or noodles. The authors state that the work provides a theoretical basis for the development of HBS and highland barley food with different expected properties. 

Takahiro Noda et al. (Contribution 3) investigated the physicochemical properties and in vitro digestibility of “Manten-Kirari” starch with trace-rutinosidase activity. The starch was analyzed by a rapid viscosity analyzer (RVA) and differential scanning calorimetry (DSC), as well as in vitro digestibility. The study finds the lowest content of amylose was observed in ‘Manten-Kirari’ starch (18.1%), while the highest was located in ‘Kitawasesoba’ starch (22.6%). The ‘Manten-Kirari’ starch exhibited a larger median granule size (11.41 µm) and higher values of peak viscosity (286.8 RVU) and breakdown (115.2 RVU) than the others. The onset temperature values for gelatinization were 60.5 °C for ‘Kitawasesoba’, 61.3 °C for ‘Manten-Kirari’ and 64.7 °C for ‘Hokkai T8′. The ‘Manten-Kirari’ and ‘Hokkai T8′ starches were digested more slowly than the ‘Kitawasesoba’ starch.

Qin et al. (Contribution 4) investigated the effects of different physical treatments on the structural and functional properties of rice flour. The treatment methods in this paper include ultrasonic treatment (US), microwave treatment (MW) and hydrothermal treatment (HT). The results of this paper show that ultrasound and microwave have little effect on the apparent morphology of starch, but hydrothermal treatment produces more fragments and cracks. Ultrasound led to a more ordered arrangement within the starch, whereas hydrothermal heat disrupted the structure of the starch. The batter with added HT exhibited the highest G′ and G″ values and the lowest tan δ. Furthermore, bread made from US and MW starch presented reduced hardness, cohesion and gumminess. According to the authors’ conclusions, it is presented that US rice flour improves the textural properties and appearance of rice bread.

Li et al. (Contribution 5) investigated the effects of gamma irradiation and annealing (ANN) on the functional properties of sago starch. In this study, the solubility and swelling power, pasting characteristics and amylose starch content of sago starch were explored using dual treatments of gamma irradiation and annealing. The final results showed that the content of amylose starch decreased under gamma irradiation and increased under ANN, and the effect was the same under dual modification as under ANN. After irradiation, the swelling power was reduced and solubility increased; after ANN treatment, both the solubility and swelling power were reduced, while the results after double treatment showed similar results to those of gamma irradiation. At the same time, neither single nor double treatment altered the integrity of starch. Based on the authors’ conclusions, gamma irradiation and ANN were able to induce some new properties of sago starch that could be used for extended applications.

Xue et al. (Contribution 6) explored the palatability, flavor, storability and starch functionality properties of different varieties of rice. In this paper, 84 varieties were selected for comparison of their various characteristics. The authors stated that the presented three YY-IJHR varieties could be identified, through their straight chain starch and protein content, as being better for cooking and consumption in relation to the N84 variety. Significant differences were seen in the pasting characteristics of the 84 rice varieties. Rice aroma components were revealed by GC-IMS, which indicated that the alcohol content of the volatile components of YY-IJHR was generally lower, whereas the content of some aldehydes and esters was higher than in N84. In terms of storage, YY-IJHR had better rice quality and storability than N84. The authors’ study clearly analyzed the variability of 84 rice varieties and is extremely helpful for both agricultural cultivation as well as food production.

Zuo et al. (Contribution 7) explored the beneficial effects of resistant starch in hyperlipidemia acute pancreatitis (HLAP). In this paper, an acute pancreatitis model was established by feeding a hyperlipidemia diet to rats and subsequently evaluating the anti-HLAP effect of RS in kidney beans. The final results show that compared with original starch, the average volume particle size of the RS granules increased significantly and the specific surface reduced significantly. Reduction in a specific surface area can avoid excessive contact between RS and enzymes, thereby enhancing its resistance to enzymatic hydrolysis. The IL-6, IL-1β and TNF-α of serum in each RS group and the depth of the crypt decreased, while the height of the villi in the small intestine and the thickness of the muscle layer of rats increased. The authors showed that resistant starch has a preventive effect on intestinal damage, which is a very interesting result for the study of human health.

## 3. Conclusions

This compilation of articles, focused on the production and functional properties of starch, includes a wide variety of research tools that illustrate the breadth of the field of study. This can be reflected in the variety of samples and starches analyzed, different extraction and modification methods, and means of detection.

In terms of the subject, starch modification has received increasing attention in recent years, with a total of six articles describing starch modification. However, it should be noted that one of the articles is focused on extraction, using processes that may make starch extraction more eco-friendly, with increased extraction rates as well as purity. Two of these articles describe the differences between varieties and are extremely beneficial to those in the food processing industry as well as in crop cultivation. The last two articles describe the beneficial effects of resistant starch on the human body, which could be helpful when considering society’s current focus on healthy eating.

In conclusion, research should not only focus on the production and properties of starch but also on exploring its structural changes, through which we can then explain the altered physicochemical and functional properties of starch. Currently, there are fewer articles on this subject in this Special Issue. Therefore, we are extremely interested in articles of this novelty, which not only provide the reader with a deeper understanding of the structural changes of starch but also allow the principles of these to be analyzed, thus enabling a more comprehensive understanding of the field of research.
